# Corneal Epithelium Expresses a Variant of P2X_7_ Receptor in Health and Disease

**DOI:** 10.1371/journal.pone.0028541

**Published:** 2011-12-06

**Authors:** Courtney Mankus, Celeste Rich, Martin Minns, Vickery Trinkaus-Randall

**Affiliations:** 1 Department of Biochemistry, Boston University School of Medicine, Boston, Massachusetts, United States of America; 2 Department of Ophthalmology, Boston University School of Medicine, Boston, Massachusetts, United States of America; 3 ECI Biotech Worcester, Massachusetts, United States of America; University of Reading, United Kingdom

## Abstract

Improper wound repair of the corneal epithelium can alter refraction of light resulting in impaired vision. We have shown that ATP is released after injury, activates purinergic receptor signaling pathways and plays a major role in wound closure. In many cells or tissues, ATP activates P2X_7_ receptors leading to cation fluxes and cytotoxicity. The corneal epithelium is an excellent model to study the expression of both the full-length P2X_7_ form (defined as the canonical receptor) and its truncated forms. When Ca^2+^ mobilization is induced by BzATP, a P2X_7_ agonist, it is attenuated in the presence of extracellular Mg^2+^ or Zn^2+^, negligible in the absence of extracellular Ca^2+^, and inhibited by the competitive P2X7 receptor inhibitor, A438079. BzATP enhanced phosphorylation of ERK. Together these responses indicate the presence of a canonical or full-length P2X_7_ receptor. In addition BzATP enhanced epithelial cell migration, and transfection with siRNA to the P2X_7_ receptor reduced cell migration. Furthermore, sustained activation did not induce dye uptake indicating the presence of truncated or variant forms that lack the ability to form large pores. Reverse transcription-polymerase chain reaction and Northern blot analysis revealed a P2X_7_ splice variant. Western blots identified a full-length and truncated form, and the expression pattern changed as cultures progressed from monolayer to stratified. Cross-linking gels demonstrated the presence of homo- and heterotrimers. We examined epithelium from age matched diabetic and non-diabetic corneas patients and detected a 4-fold increase in P2X_7_ mRNA from diabetic corneal epithelium compared to non-diabetic controls and an increased trend in expression of P2X_7_variant mRNA. Taken together, these data indicate that corneal epithelial cells express full-length and truncated forms of P2X_7_, which ultimately allows P2X_7_ to function as a multifaceted receptor that can mediate cell proliferation and migration or cell death.

## Introduction

Wound closure and response to disease involve a series of complex biologic events. Rapid and long-term signals are generated in response to the release of nucleotides from cells upon cell stress, mechanical stimulation, ligand binding or injury. The stimulation can lead to an increase in cytosolic calcium generated by the activation of purinergic receptors (P2Y or P2X) through distinct mechanisms. The P2Y receptors are G-protein coupled, while the ionotropic P2X receptors form cation channels and allow for the influx of extracellular Ca^2+^ [1], [2]. Both are present in corneal epithelial cells where the distinct mechanisms of increasing intracellular Ca^2+^ are displayed [3]. For example stimulation of the P2Y receptors attenuates the injury induced calcium wave, while after activation of the P2X_7_ receptor with BzATP it is not diminished [3].

The canonical P2X_7_ receptor (defined as full-length receptor) contains an extended C-terminus compared to the other P2X receptors [4]. While the protein was initially hypothesized to be a cell death inducing receptor, it is now implicated in both proliferative and death processes, which reflect the expression of the truncated or full-length receptor respectively. One of the features of the full-length receptor is its ability to induce the formation of large non-selective pores in the cell membrane in response to prolonged or repeated stimulation. The non-specific pore allows for the passage of organic cations up to 900 Da into the cell and can be monitored by the uptake of fluorescent dyes [4]–[8]. In addition many of the functions of the canonical P2X_7_ receptor such as membrane blebbing, formation of large pores and a cytotoxic response depend on protein interactions with specific domains in the C-terminus [9]–[11]. In contrast the lack of pore formation, dye uptake and cytotoxicity is defined as non-canonical activity and attributed to P2X_7_ splice variants [10]–[12].

The cell death induced by activation of the canonical receptor occurs via import of lethal intracellular Ca^2+^ levels, activation of caspases, and ultimately apoptosis or necrosis [13]–[18]. In this regard the P2X_7_ receptor is implicated in inflammation and is expressed in immune cells and cells of the central nervous system [13], [19] where it is a mediator of a number of interleukins that may serve to integrate the response [5], [17], [20], [21]. This is supported by data demonstrating that P2X_7_^−/−^ mice are resistant to inflammation [22]. However, its activation has been shown to yield a number of other activities including the growth of human neuroblastoma cells [23], the increase in mitogenic activity in peripheral blood lymphocytes [20] and enhanced expression in cervical cancer [11], [12]. In addition, activation of the receptor has been shown to result in the phosphorylation of MAP kinase [25]–[27]. Still other work has shown that epithelial adhesion to basal lamina and corneal wound repair in P2X_7_^−/−^ mice is attenuated [28]. These data indicate that the receptor can play several different roles that may be attributed to receptor variants.

In this study, we show that corneal epithelial cells display both canonical and non-canonical responses. BzATP induces a canonical slow onset Ca^2+^ wave, which is absent in Ca^2+^ free media and is attenuated in the presence of Zn^2+^ or Mg^2+^. In contrast, cell migration is enhanced with BzATP, and reduced significantly in cells transfected with siRNA to the P2X_7_ receptor. Furthermore cytotoxic responses and dye uptake were not detected in confluent cultures upon P2X_7_ activation, which correlates with the expression of the P2X_7J_ variant mRNA and the expression of both 75 and 42 kDa forms. However, in stratified cell cultures there is a decrease in the expression of the truncated form, which correlates with uptake of ToPro-3 in the most apical cells. In addition we demonstrate that the P2X_7_ mRNA is increased in diabetic corneal epithelium compared to control. Together these data indicate that the expression of full length and truncated forms allows P2X_7_ to function as a multi-faceted receptor.

## Materials and Methods

### Ethics Statement

Healthy and diabetic human donor corneas were purchased from National Disease Research Interchange (NDRI) with permission from Cedars-Sinai Medical Center IRB Protocol EX-1055. NDRI has a human tissue collection protocol that is subject to National Institutes of Health oversight.

### Materials

Keratinocyte serum-free culture medium was obtained from Invitrogen (Carlsbad, CA), DMEM was from Mediatech, Inc. (Manassas, VA) and fetal bovine serum (FBS) was from Gemini bio-products (West Sacramento, CA). Telomerase immortalized human corneolimbal epithelial (HCLE) cells were a kind gift from Dr. I. K. Gipson, Schepens Eye Research Institute, Harvard University (Boston, MA), and IMR90 fibroblasts were from ATCC (Manassas,VA). The P2X_7_ antibody was purchased from Alomone Labs (Jerusalem, Israel). Anti-fade, Fluo-3/AM, pluronic acid, propidium iodide, and To-Pro 3 were purchased from Invitrogen. Secondary antibodies (HRP-conjugated goat anti-rabbit and donkey anti-goat), and protein A agarose were from Santa Cruz Biotechnology (Santa Cruz, CA). PNGase F was from New England Biolabs (Ipswich, MA), and the BCA Protein Assay kit was purchased from Pierce (Rockford, IL). Western blot chemiluminescence reagent and PVDF membranes were from Perkin Elmer (Boston, MA). The Oligotex mRNA kit and DNeasy kit were purchased from Qiagen (Valencia, CA), and the Fluorescent Caspase-3 Assay kit was from Biotium (Hayward, CA). The siRNAs directed against P2X_7_ and P2Y_2_ were from Dharmacon (Lafayette, CO). Lipofectamine 2000, anchored oligo-[DT]_20_ primer, TRIzol, DNase I, random hexamers, M-MLV reverse transcriptase, and RNase H were purchased from Invitrogen, and the master Taq kit for RT-PCR was from Fisher Scientific (Pittsburgh, PA). TaqMan probes and TaqMan gene expression master mix for real time RT-PCR were obtained from Applied Biosystems (Foster City, CA). A 438079 hydrochloride was purchased from Tocris Bioscience (Ellisville, MO). For northern blot analysis, [^32^P]dCTP was purchased from Perkin Elmer (Boston, MA). Nytran® membrane was from Schleicher and Schuell Bioscience, Inc. (Keene, NH), the nick translation system was from Invitrogen, and autoradiographic film was from Kodak (Rochester, NY). Adenosine triphosphate (ATP), 3′-O-(4-benzoyl) benzoyl adenosine 5′-triphosphate (BzATP), ethidium bromide were purchased from Sigma Aldrich, Inc. (St. Louis, MO) and other routine chemicals were obtained from American Bioanalytical (Natick, MA).

### Cell culture

HCLE cells were cultured in supplemented keratinocyte serum-free medium (K-SFM) [29]. Twenty-four hours prior to all experiments, EGF and BPE were removed. The response of HCLE cells to injury and Ca^2+^ mobilization is similar to primary epithelial cultures [3], [30]. To stratify the cells, cultures were maintained until they achieved confluence and the media was switched to DMEM:Ham's F-12 (1∶1) containing elevated Ca^2+^ supplemented with FBS and EGF [31]. IMR-90 fibroblasts were cultured in Eagles Minimal Essential Medium containing 10% FBS according to the manufacturer's protocol (ATCC).

### Calcium imaging

Once cells achieved confluence, Ca^2+^ imaging was performed as previously described [29]. Experiments were performed either in complete HEPES-buffered saline, Ca^2+^-free HEPES-buffered saline, or in Mg^2+^-free HEPES-buffered saline. Cells were perfused with HEPES-buffered saline prior to stimulation to establish a base line fluorescence. Perturbations were made while scanning the cells every 789 ms. Cells were stimulated with agonists prepared in HEPES-buffered saline and washed with HEPES-buffered saline and Ca^2+^ dynamics were evaluated [29], [32]. A438079 was included in the fluo-3 solution during the 20 min incubation prior to experimentation as well as in all flow through solutions.

### Reverse transcription-polymerase chain reaction (RTPCR)

Messenger RNA was isolated from corneal epithelial cells using the Oligotex mRNA Kit according to manufacturer's directions. mRNA was annealed with anchored oligo–[DT]_20_ primer and first strand cDNA synthesis was carried out with M-MLV reverse transcriptase. Primers to P2X_7_ were designed to anneal to consensus sequences found in all of the known P2X_7_ variants and are as follows: forward primer: 5′-ACAGGAAGAAGTGCGAG TCC-3′, reverse primer: 5′-GGTAGAGCAGGAGGAAC TGC-3′. Additional primers were designed to specifically amplify P2X_7_ variants f and j as both of these variants contain the same frameshift mutation in exon 8: forward primer: 5′-TTTCAGATGTGGCAATTCAGATA-3′, reverse primer: 5′- AAGTAGGAG AGGGTTGAGCC -3′ [12]. DNA amplification was carried out for 45 cycles (30 s 95°C, 30 s 60°C, 1 min 72°C) followed by 10 min at 72°C. The amplified PCR products were examined on 1.2% agarose gels stained with ethidium bromide and specificity was confirmed by sequencing performed by Invitrogen. For amplification of specific P2X_7_ exons, genomic DNA was extracted from HCLE cells using the DNeasy kit according to manufacturer's instructions. PCR was performed using the primers listed in Table 1.

**Table 1:** 

Target	5′-Forward primer-3
	5′-Reverse Primer-3′
Exon 1	TCAGAATGTGCACCTGAAGC
	CCAGTACGTTTCATTTTGCAG
Exon 2	GGCTGTAGATCCTAGGGGAAG
	AGTCACACGGAAGCAAGTCA
Exon 3	GTCCGCATTTCTGCTTCTTC
	CCCAGCAAGCTGGATTATTA
Exon 4	TGACCTGGGCATCACAAAT
	GTGTGCACATTCTGGTGGAT
Exon 5	TAGGACCCAGGACTTTGCAG
	CGGGTTGAGTTAATGATGTCC
Exon 6	TTCAGGCTTCTGAGGTTTGG
	AGAAGCCTCTGGTCCCACTG
Exon 7	GCCTCTTGGCTGTTTGACAT
	TGGAACCTCTCCACCACACT
Exon 8	GTTGCCTTGGAAACCAAAAT
	CTATGCAGGGAGATGTCTGG
Exon 9	GCCCCACAGCAGTAATTAGG
	GCTGCAGTGAGTGGTAATCCT
Exon 10/11	TAGAACCCAGCGACGTATCC
	CCAACAATTGCACGTTGAAG
Exon 12	GGGGCATAAAAGGGACTCCT
	TGAGCCAGCTTGTTCAATAGTC
Exon 13	CAGACGTGAGCCACGGTGC
	GAACCTAGAACCTGAGGGCT

### Real time RT PCR

Cells were lysed in TRIzol and RNA was extracted according to manufacturer's guidelines. Genomic DNA was removed by incubation with DNase I in the presence of 1 unit/ml RNase inhibitor. RNA was annealed with random hexamers and first strand cDNA synthesis was carried out with M-MLV reverse transcriptase. Negative control runs were performed without the reverse transcriptase. The cDNA was treated with RNaseH. Real time RT-PCR was performed using an ABI 7300 (Applied Biosystems). The TaqMan^®^ gene expression assays used were: Hs00175721_m1 for P2X_7_, Hs00602525_m1 for P2Y_2_, Hs00267404_s1 for P2Y_4_, Hs00602442.m1 for P2X_4_, Hs01032443-m1 for Ki67 and the Eukaryotic 18S rRNA Endogenous Control (Vic/MGB Probe, Primer Limited Applied Biosystems). For P2X7J primers described above in RT-PCR, ABI Sybr green master mix was used. The cycling parameters were as follows: 50°C 2 min, 95°C 10 min, 45 cycles of 95°C 15 s and 60°C 1 min. Results are presented as relative expression normalized to 18s rRNA and were calculated using the ΔΔCt method.

### Northern blot analysis

Total RNA was extracted from HCLE cells using 4 M guanidinium thiocyanate as described [33]. Messenger RNA was isolated by passing the total RNA twice over an oligo-dT column to ensure complete removal of rRNA. RNA was resolved by electrophoresis through a 1.2% agarose gel containing 6% formaldehyde and transferred to a Nytran® filter. Lack of rRNA contamination in the purified mRNA was confirmed by soaking the filter in 0.04% methylene blue in 0.5 M N-Acetate, pH 5.2 for 10 min. A 412 bp fragment of P2X_7_ amplified by PCR was used as it picked up the canonical form and all variants. The primers used for amplification are directed against a consensus sequence described above. The probe was labeled with [^32^P]dCTP by the nick translation method according to manufacturer's protocol. The hybridization proceeded at 42°C in a solution containing 0.5% SDS, deionized formamide, 5 mM EDTA, 0.9 M NaCl, 50 mM Na_2_HPO_4_ (pH 7.0), and the probe. After hybridization, the blots were washed twice in 5x SSC, 1% SDS at 37°C for 30 min, followed by twice in 1X SSC, 1% SDS at 37°C for 30 min and once in 0.1X SSC, 0.1% SDS for 15 min at 60°C and subjected to autoradiography.

### SDS PAGE and Western Blot Analysis

Cells were cultured for 18–24 h before experimentation in K-SFM lacking growth factors. Cells were either treated with 100 µM BzATP in K-SFM lacking growth factors for specified times or lysed without treatment as indicated in figure legends. Cells were rinsed with cold phosphate buffered saline (PBS, pH 7.4), lysed in 10 mM Tris-HCl (pH 7.4) containing 1% Triton X-100, 0.5% Nonidet P-40, 150 mM NaCl, 1 mM phenylmethylsulfonyl fluoride (PMSF), and 1 mM sodium orthovanadate (Na_3_VO_4_), and sheared using a 20G needle. Total protein concentration was determined using a bicinchonic acid (BCA)-based method. Equivalent amounts of protein (40 µg) from each sample were subjected to SDS-PAGE and transferred to a nitrocellulose membrane by the semi-dry method. Nonspecific binding was blocked with 5% milk in a PBS buffer (137 mM NaCl, 2.7 mM KCl, 10 mM phosphate, 0.1% Tween-20) according to Alomone. Membranes were probed with primary antibody (1∶200), washed, and incubated with horseradish peroxidase-conjugated secondary antibody. The chemiluminescence enzymatic reaction was carried out (Denville Scientific, Inc., Metuchen, NJ).

### In situ Crosslinking

Cells were cultured until confluent and cross-linking was performed in situ after media was replaced with 10 ml of media without additives. 270 µl of 37% formaldehyde was added dropwise with swirling to make a 1% solution for 10 minutes at room temperature. Glycine (1.0 ml of 1.25 M) was added to quench the unreacted formaldehyde, swirled and incubated for 5 minutes. Cultures were placed on ice and rinsed twice with ice cold PBS, scraped in 1.0 ml cold cell lysis buffer [PBS containing sodium vanadate (0.2 mM) and phenylmethanesulfonyl fluoride (1mM)] and transfered to a 1.5 ml centrifuge tube. The cell suspension was spun at 3,000 RPM for 3 min and the pellet was resuspended in cell lysis buffer and frozen at –20°C. Control samples were prepared the same way but did not contain formaldehyde. Protein concentration was determined by BCA and an equivalent amount of protein (50 µg) was subjected to SDS-PAGE and transferred for immunoblotting. To maintain the crosslinks, the sample was heated at 65°C for 5 min while disruption of the crosslinks was performed by heating samples to 95°C for 20 min in SDS sample buffer.

### Dye uptake experiments

HCLE cells were cultured in 8-well glass chamber slides or on slide coverglass. Slides were placed on the heated stage of a Zeiss LSM 510 confocal microscope and maintained at 37°C and 5% CO_2_ using the Zeiss Environmental Chamber. Cells were treated with control media, ethidium bromide (EtBr) or ToPro-3 alone in the presence of 100 µM BzATP. Fluorescent and differential contrast images were taken continuously (every 789 msec/frame) for 20 min. The excitation and emission wavelengths were as follows: for EtBr, ex_543 nm_, em_605 nm_, and for ToPro-3, ex_633 nm_ and em_661 nm_. Cultures were monitored for membrane blebbing and dye uptake.

### Cytotoxicity assays

Corneal epithelial cells were cultured to 80% confluence in 8-well glass chamber slides and incubated for 18–24 hr before experimentation in K-SFM lacking growth factors. Cells were treated with BzATP (100 µM), actinomycin D **(**2 µg/ml), or control media for 15 min, 4 hr, or 20 hr. The NucView TM 488 caspase-3 substrate (ex _488 nm_, em _530 nm_) was resuspended in unsupplemented K-SFM to a final concentration of 5 µM. After the treatment, the media was replaced with the caspase-3 substrate solution and cells were incubated further for 15 min at room temperature prior to imaging according to manufacturer's directions (Biotium, Hayward, CA). Fluorescence was visualized on the Zeiss LSM 510 200M microscope. For the MTT assay, MTT reagent was added for an additional 2 hrs following treatment with BzATP or actinomycin D, formazan was solubilized in 0.1 N HCl in isopropanol, and absorbance at 570 nm was measured. Absorbance at 690 nm was measured as background; final readings are 570 nm minus 690 nm.

### Chemotactic migration assay

The transwell migration assay was performed as described [3], [38]. Migration was performed at 37°C and 5% CO_2_ for 8 hours. Migrated cells were fixed with methanol, stained with 5 µg/ml propidium iodide and membranes were mounted onto glass slides. For each membrane (33.2 mm^2^), the total numbers of cells were counted in 6 random 10x fields (one field  = 1.37×1.08 mm, or 1.48 mm^2^) and an average and standard error were calculated.

### siRNA transfection

HCLE cells were transfected at 30–50% confluence, 24 hr following passage. Prior to addition of transfection reagents, complete media were replaced with K-SFM lacking antibiotics and supplements (transfection media). The siRNA sequences directed against P2X_7_ were sense: 5′- GGAUCCAGAGCAUGAAUUAUU -3′, anti-sense: 5′- UAAUUCAUGCUCUGGAUCCUU -3′ and against P2Y_2_: sense: 5′- GAACUGACA GCAGAGGAUUU -3′, anti-sense: 5′- AUCCUCUGCAUGUCAGUUCUU -3′. A final concentration of 100 nM siRNA or an equal concentration of non-targeting siRNA (Dharmacon product # D-001206-13-20) was transfected using 2 mg/ml Lipofectamine 2000 according to manufacturer's protocol. The transfection media and reagents were replaced with complete K-SFM 6 hr post transfection, and the cells were cultured for 4 days prior to experimentation, which was optimized previously [30].

### Scratch wound assay

Corneal epithelial cells were passed into 8-well glass chamber slides and transfected with either P2X_7_ siRNA or a non-targeting control siRNA as described and imaged as described by [29], [30], [32], [34], [35]. Briefly, cells were incubated at 37°C and 5% CO_2_ using the Zeiss environmental chamber for 20 h. Two wounds were made per well and cells were incubated in the presence or absence of BzATP. After 20 h, the images were concatenated using the Zeiss LSM 510 Multi-Time software, and the LSM 510 software was used to measure changes in the wound area over time. Percent change in wounded area was calculated. Repeats were performed within runs and between runs to assess consistency and representative data are shown. Statistical comparisons were made using Student's t-test or ANOVA followed by Tukey's post hoc test.

### Human tissue

Healthy and diabetic human donor eyes and corneas were purchased from National Disease Research Interchange (NDRI, Philadelphia, PA), under Cedars-Sinai Medical Center IRB protocol EX-1055 (see Ethical Statement). Upon receipt, central corneal buttons were cut out with an 8.25-mm trephine, epithelium removed and tissue immediately frozen in liquid nitrogen and RNA isolated. A total of 12 corneas were used (6 non-diabetic, mean age 69y and 6 diabetic, mean age 75y).

### Statistics

Statistical comparisons were made using linear regression, Student's t-test or Analysis of Variance (ANOVA) followed by Tukey-Kramer posthoc test or Tukey posthoc test.

## Results

### BzATP induces mobilization of extracellular Ca^2+^ -a canonical activity of the P2X_7_ receptor

**Figure 1:**
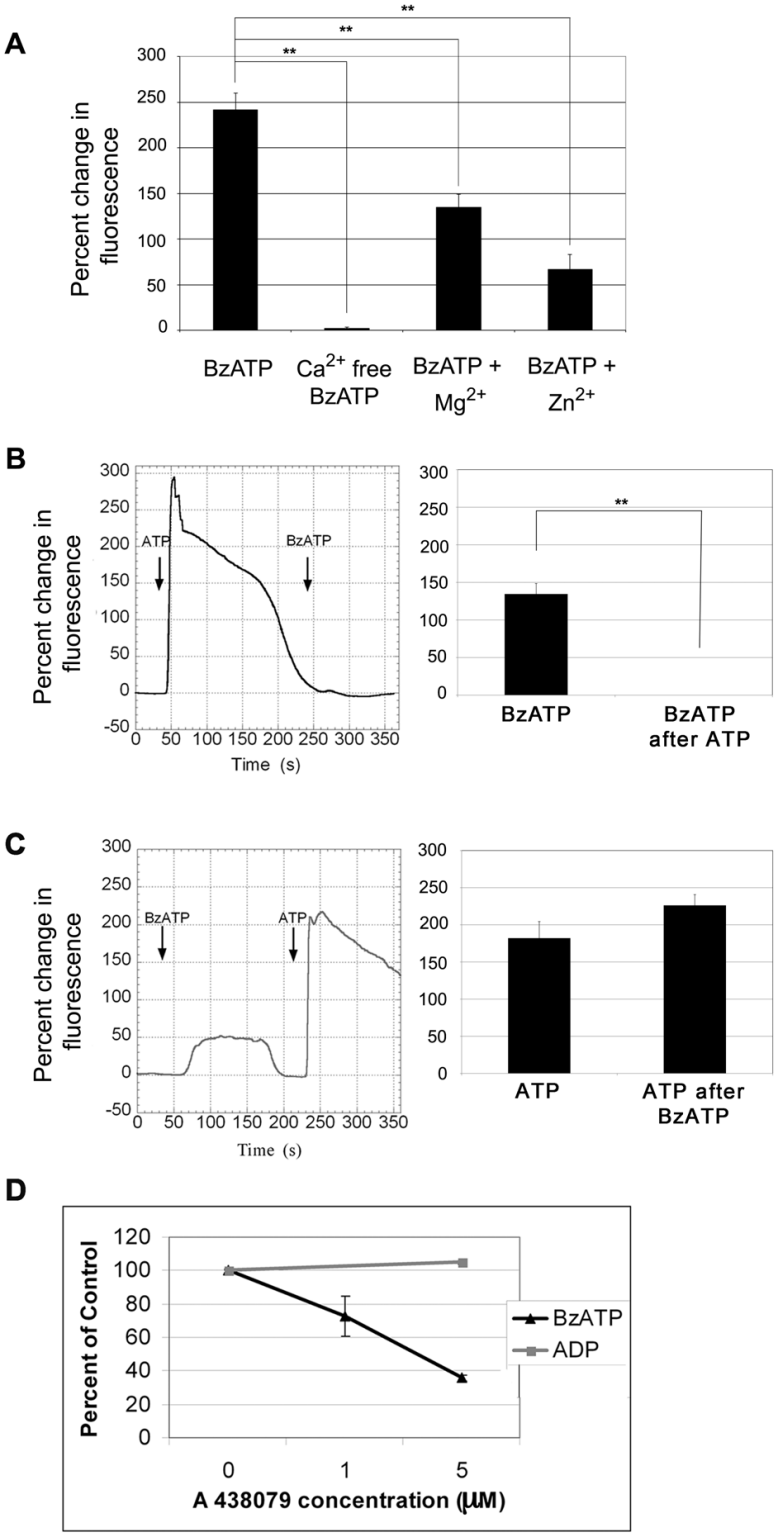
BzATP causes an increase in intracellular Ca^2+^. HCLE cells were incubated in 5 µM fluo-3AM for 30 min and imaged in a flow-through apparatus on a Zeiss LSM 510 confocal microscope. Cells were washed with indicated HEPES-buffered saline (control, Ca^2+^ free, 4 mM Mg^2+^, 100 µM ZnSO_4_) and stimulated with BzATP in corresponding buffer for 2 min. Maximal percent change in average fluorescence of a 460 µm×460 µm field was determined. **A.** HCLE cells stimulated with BzATP in HEPES buffer, in Ca^2+^ free - HEPES buffer, in HEPES buffer with Mg^2+^ and in HEPES buffer with Zn^2+^. Graphs represent a minimum of six independent experiments +/− SEM. Analysis of variance was determined using the general linear model procedure followed by Tukey-Kramer posthoc test. **p<0.001. **B**. Response of HCLE cells to ATP followed by stimulation with BzATP. Representative trace of changes in fluorescence over time. Graph represents difference between stimulation with ATP and BzATP after ATP and represents a minimum of six independent experiments +/− SEM. Students t-test ** p<0.001. **C** Response of HCLE cells to BzATP followed by ATP. Representative trace of changes in fluoresence over time. Graph represents difference between stimulation with BzATP and ATP after BzATP and represents a minimum of six independent experiments +/− SEM. **D.** Cells were washed with HEPES buffered saline with the indicated concentrations of A438079 and stimulated with BzATP or equimolar concentrations of ADP for 2 min in the presence or absence of inhibitor. The average maximum percent change in fluorescence is graphed as a percentage of the uninhibited control +/− SEM. Each time point is an average of 12 independent experiments. Linear regression analysis where the slope of the line for BzATP is significantly different from 0. p = 0.0001. The control ADP is not significantly different from 0.

Previously we demonstrated that confluent corneal epithelial cells responded to BzATP and determined the EC_50_ for BzATP in human corneal epithelial cells to be (8.9±5.9) ×10^−5^ M using live cell Ca^2+^ imaging [3]. To perform these experiments, cells were loaded with Fluo-3AM, were stimulated with a constant flow of agonist in HEPES buffered saline. Change in fluorescence was measured relative to the basal level [3]. The flow rate was set so that HEPES alone did not stimulate any response. We compared the response to BzATP under different ionic conditions. When cells were stimulated with 100 µM BzATP, there was a significant increase in fluorescence (Figure 1A). In contrast, when experiments were performed in a calcium-free HEPES buffer, there was a minimal increase in fluorescence in response to BzATP (1.83%) (Figure 1A). This result indicates that the response is the result of calcium influx from the extracellular milieu and not the result of P2Y activation and release of calcium from intracellular stores [3]. In the presence of extracellular Mg^2+^ the increase in fluorescence was reduced from 241.5±18.3% to 134.6±14.2% and in the presence of extracellular Zn^2+^ the response was attenuated further (66.6±16.7%) (Figure 1A). These changes observed in the presence of extracellular cations are considered a hallmark of P2X_7_ activation and demonstrate canonical activation [4], [36]–[38]. Additional Ca^2+^ imaging experiments were performed in which cells were stimulated with BzATP or ATP for 2 min, washed with HEPES-buffered saline for 2 min to restore intracellular Ca^2+^ levels back to baseline and then stimulated with a second agonist for 2 min. When HCLE cells were stimulated with ATP followed by BzATP there was a significant diminished response to the second stimulus compared to BzATP alone. However, when the treatments were reversed, BzATP did not desensitize the ATP response (Figure 1B, C). When cells were repeatedly stimulated with BzATP there was no desensitization compared to stimulation with ATP, UTP or ADP [38]. Together these data support the evidence for canonical receptor activity.

The Ca^2+^ response elicited by BzATP was inhibited by A438079, a highly selective competitive P2X_7_ inhibitor in a dose dependent manner (Figure 1D). The IC_50_ of the inhibitor is 300 nM for human receptors and concentrations 50 fold greater were used [39]. At 1 µM, A438079 inhibited the response by 27.4%, and at 5 µM a 64.2% inhibition was achieved. Higher concentrations were not used due to decreased specificity with inhibition of pannexin-1 observed at 10 µM [40]. To confirm the specificity of the inhibitor the response to ADP, a ligand for P2Y_1_ receptor, was examined. As expected there was no change in the average percent increase in fluorescence upon stimulation with ADP in the presence of 5 µM A438079 (Figure 1D).

### Activation of P2X_7_ receptor mediates ERK1/2 phosphorylation indicating presence of the amino terminus

**Figure 2:**
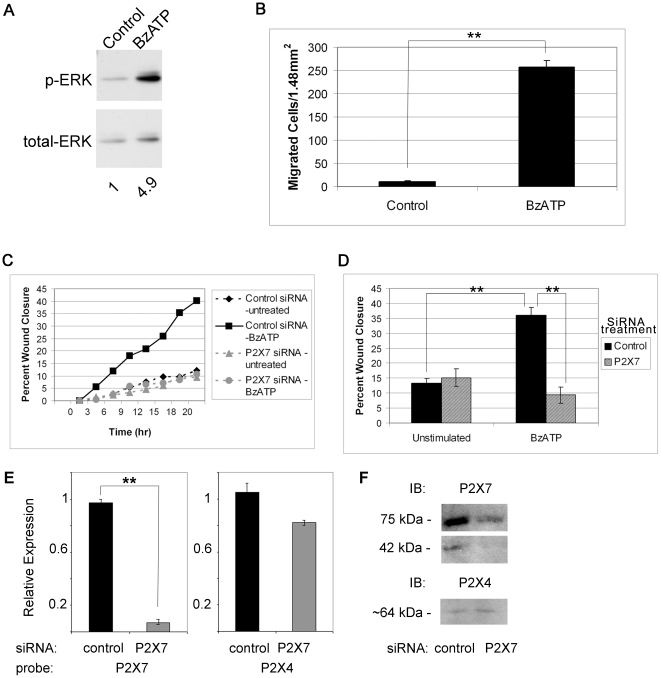
P2X_7_ activation induces phosphorylation of ERK and cell migration. **A.** Cells were treated in the presence or absence of BzATP for 5 min, lysed and equivalent amounts of protein were resolved by SDS-PAGE and immunoblotted with anti-p-ERK. Blots were stripped and reprobed with anti-ERK and pERK normalized to ERK. Significance was determined by Student's test (p<0.05). Blot is representative of 3 independent experiments. **B.** Transwell migration assays were performed for 8 hr in the presence of BzATP or binding buffer (control). Migrated cells were stained with propidium iodide, counted in 6 randomly chosen fields (1.48 mm^2^) and averaged. Data are representative of 3 independent experiments and are presented as mean +/− SEM. Significance was determined by Student's t-test: **p<0.001. **C.** Downregulation of P2X_7_ attenuates wound healing in a representative experiment. HCLE cells transfected with control (non-targeting siRNA) or siRNA targeted to P2X_7_ receptor were cultured to confluence. Unsupplemented media or media with 100 µM BzATP were added prior to injury. Slides were placed on a heated stage in an environmental chamber at 37°C and 5% CO_2_. Scratch wounds were made and contiguous images were taken every 20 min over 20 hr. Data are representative of 3 independent experiments. **D**. Percent wound closure from directed migration experiments at endpoint are presented as mean +/− SEM. Significance was determined by ANOVA followed by Tukey posthoc test: **p<0.001. **E.** HCLE cells transfected with control (non-targeting siRNA) or siRNA targeted to P2X_7_ receptor were cultured to confluence. Real time RT-PCR was performed and relative expression of the indicated receptors was determined using the ΔΔCT method. The average relative expression of 3 independent experiments +/− SEM is presented. Significance was determined by Student's test: **p<0.004. **F.** Parallel experiments to **E**. were conducted and cultures were lysed and equivalent amounts of protein resolved by SDS-PAGE and immunoblotted with antibodies directed to P2X_7_ and P2X_4_ receptors.

Previously we showed that ATP is released by mechanical injury and leads to ERK phosphorylation in a rapid and transient manner. Furthermore, phosphorylation was attenuated when the wound medium was supplemented with apyrase [41]. Since the amino terminus of P2X_7_ contains the domain required for ERK activation, cells were stimulated with BzATP and phosphorylation of ERK was examined. When corneal epithelial cells were treated with 100 µM BzATP a 4.9-fold increase in phosphorylation of ERK was detected at 5 min compared to control (Figure 2A). The minimum increase observed at 5 min was 4-fold with a maximum of 10 fold, which compared to stimulation with other agonists and wound media at 5 min [41].

### Cell migration requires activation of P2X_7_ receptor

As we had previously shown that wound repair was impaired in P2X_7_^−/−^ mice [28], we asked if activation of the receptor could induce cell motility and wound closure of epithelial cells. The first set of experiments addressed whether BzATP acted as a potent chemoattractant. Assays were performed at concentrations previously shown to optimize motility [3]. In a representative experiment, 258 cells per 1.48 mm^2^ migrated towards BzATP (10 µM) compared to only 11 cells per 1.48 mm^2^ in the control binding buffer (Figure 2B). Scratch wound migration assays were performed to address the role of BzATP and the P2X_7_ receptor on wound repair in vitro. HCLE cells plated on chambered glass slides were transfected with siRNA directed against P2X_7_ or non-targeting (control) siRNA and grown to confluence. Scratch wounds were made resulting in a cell-free area, and closure of the wound was monitored every 20 min over a period of 20 hr on a Zeiss LSM 510 200M confocal microscope with a heated stage and CO_2_ regulated chamber. Previously we established a wound size for which proliferation was not required to close the wound [35]. Wound closure in cells transfected with control siRNA or siRNA directed against P2X_7_ did not differ after 20 hr (Figure 2D). The addition of BzATP enhanced the average wound closure significantly in cells transfected with non-targeting siRNA. There was no increase in cells transfected with P2X_7_ receptor siRNA and stimulated with BzATP (Figure 2C and D). These indicate that the cells transfected with siRNA to the P2X_7_ did not respond to the agonist, while the cells transfected with the non-targeting siRNA responded to the BzATP and migrated. Real time RT-PCR was performed and showed significant knockdown of the P2X_7_ receptor (Figure 2E). In addition western blot analysis showed that the 75 kDa form was 12.3% of the non-targeting control and the 42 kDa form was 12.5% of the non-targeting control (Figure 2F). As P2X_4_ has been shown to be co-expressed with P2X_7_, expression of P2X_4_ mRNA and protein in cells transfected with siRNA to P2X_7_ was evaluated. There was no significant decrease in mRNA or protein expression (Figure 2E, F **)**.

### P2X_7_ receptor activation by BzATP does not mediate cytotoxicity: a canonical activity of the receptor

**Figure 3:**
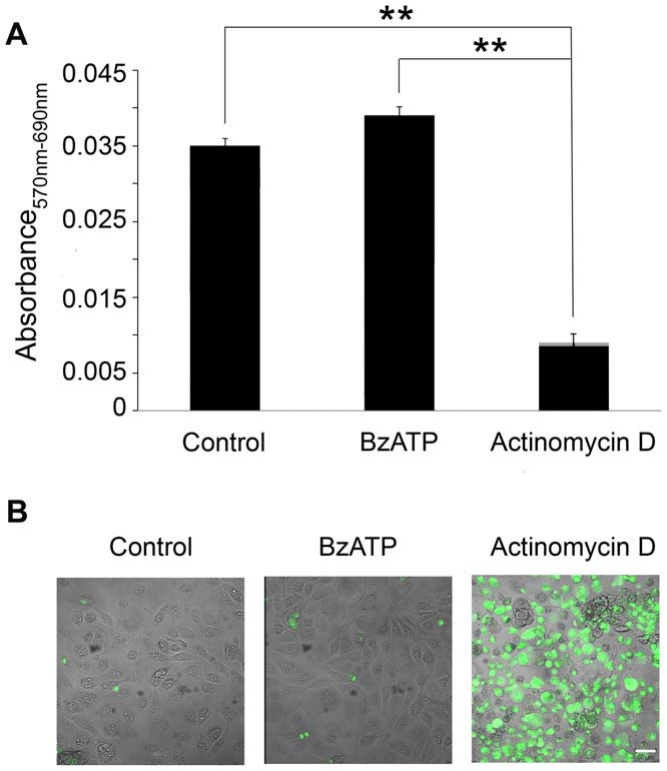
Cytotoxicity is not induced in epithelial cells following P2X_7_ activation. **A.** MTT assay for cell toxicity was performed on confluent HCLE cells. The response to control media, 100 µM BzATP or actinomycin D is presented as corrected absorbance (570nm–690nm). Data are representative of 3 independent experiments and are presented as +/− SEM. (Significance was determined by ANOVA followed by Tukey posthoc test; ** p<0.001. **B.** HCLE cells were stimulated with 100 µM BzATP, 2 µg/ml actinomycin D or control media lacking growth factors for 20 hr. Caspase activation was detected with NucView 488 Live Cell Caspase-3 Assay (scale bar  = 50 µm and applies to all 3 images). Images are representative of 5 independent experiments.

The results presented thus far indicate that the mobilization of Ca^2+^ following stimulation with BzATP is canonical. However, the ability of BzATP to stimulate wound repair and cell migration instead of cell death is indicative of a non-canonical P2X_7_ response. Therefore, we examined the ability of BzATP to induce a cytotoxic response in corneal epithelial cells using the cell viability MTT assay. Confluent cells stimulated with BzATP showed no detectable reduction in viability compared to untreated controls. When the experiment was performed in the presence of actinomycin D for 20 hr, there was an 80% reduction (Figure 3A). To further examine the ability of BzATP to induce a cytotoxic response, the cleavage of caspase 3 was analyzed. Treatment with 100 µM BzATP for 5 min, 10 min, 15 min, 4 hr, or 20 hr did not induce cleavage of caspase 3 significantly over control. Actinomycin D (2 µg/ml) was used as a positive control and induction of caspase 3 was observed 20 hr following treatment **(** Figure 3B). Confirmatory experiments performed under similar conditions demonstrated lack of DNA ladder degradation (data not shown). These data suggest that the non-canonical response may indicate that the receptor is expressed as a variant form in these cells.

### Large molecule dye uptake upon BzATP stimulation does not occur in epithelial monolayer cultures

**Figure 4:**
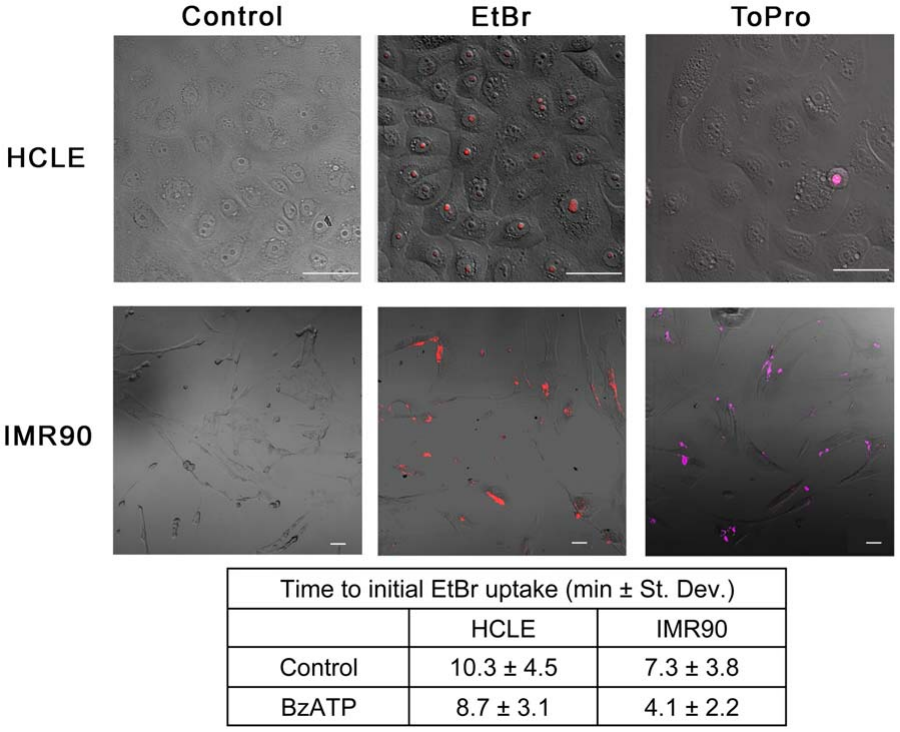
Confluent monolayer corneal epithelial cells do not form large pores when stimulated with BzATP. HCLE or IMR90 cells were cultured and stimulated with control media or media containing 100 µM BzATP in the presence of EtBr or ToPro-3 and imaged over 20 min while maintained at 37°C and 5% CO_2_ in an environmental chamber on a Zeiss LSM 510 confocal microscope. Representative images are of cells after 20 min BzATP with EtBr (EtBr) or BzATP with ToPro-3 (ToPro)(Scale bar  = 50 µm). Control treated cells in the presence of EtBr are shown (Control). ToPro-3 is detected in IMR90 cells and only detected in HCLE cells being shed. The time to initial EtBr uptake is presented. Images are representative of 3 independent experiments.

Another canonical activity associated with the P2X_7_ receptor is the ability of BzATP to cause formation of large pores. Specifically, pore formation has been linked to the C-terminus of P2X_7_ [10], [11]. To determine if pore formation occurred, HCLE cells were stimulated and uptake of ethidium bromide (EtBr) a low molecular weight dye (394 Da) or ToPro-3 a high molecular weight dye (671 Da) was examined over time. Uptake of EtBr was determined in confluent cultures using live cell microscopy at 37°C and 5% CO_2_ in the presence and absence of BzATP (Figure 4). When cells were stimulated with BzATP, uptake of EtBr was observed in 67% of HCLEs and 60% of IMR90 cells. However, initial uptake by IMR90 cells occurred more rapidly (Figure 4 and associated Table). Furthermore when IMR90 cells, a cell line known to undergo apoptosis [42], were stimulated with BzATP65.4% of the cells took up ToPro-3 (671 Da) (Figure 4). If HCLE cells were indeed forming a large pore, uptake of ToPro-3 (671 Da) would be expected in response to BzATP and it was not observed in equivalent monolayer cultures (Figure 4). Epithelial cells were also examined for membrane blebbing during dye uptake experiments, as this is another hallmark characteristic of canonical P2X_7_ receptor activity attributed to the carboxyl terminus [43]–[45]. We found that as long as the experiments were performed in an environmental chamber at 37°C and 5% CO_2_ there was no indication of membrane blebbing in response to BzATP in the live cell images and micrographs of epithelial cells (Figure 4). Together these data suggest that the P2X_7_ receptor may be expressed as a variant form in epithelial cells and that the expression may be cell-type specific.

### Expression of P2X_7_ receptor full-length and truncated forms by epithelial cells

**Figure 5:**
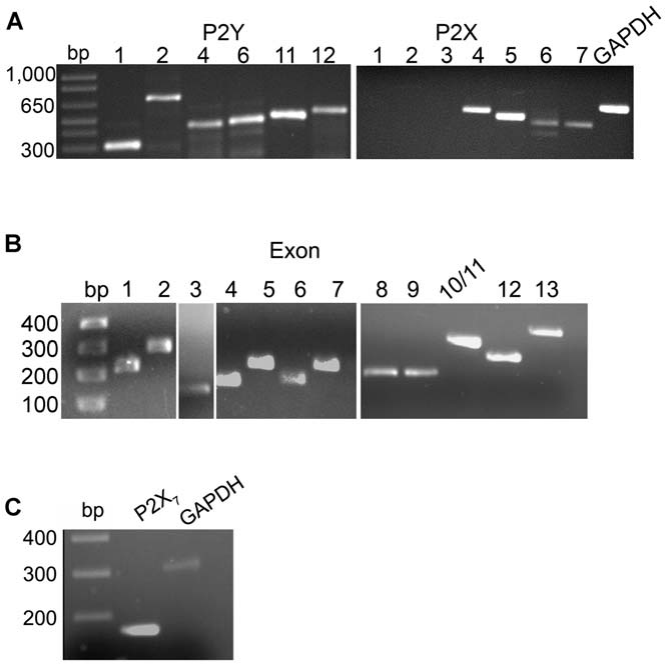
Expression of receptor transcripts in epithelial cells. **A.** RT-PCR was performed on HCLE mRNA showing presence of P2Y 1, 2, 4, 6, 11, and 12 receptor transcripts and P2X 4, 5, 6, and 7 receptor transcripts. GAPDH is included as internal control. **B.** PCR was performed on genomic DNA using exon specific primers. **C.** RT-PCR was performed on HCLE mRNA using primers that spanned exon 8 (to amplify variants f and j), showing an expected product size of 169 bp. GAPDH was amplified as an internal control. All products were sequenced and verified. Data is representative of a minimum of 3 independent experiments.

As the response to BzATP indicated the presence of both canonical and non-canonical P2X_7_ receptor induced activity in corneal epithelial cells, the expression profile of P2Y and P2X receptors was determined. RT-PCR revealed the presence of P2Y receptors (1, 2, 4, 6, 11 and 12) and P2X receptors (4, 5, 6 and 7 but not 1, 2, or 3) (Figure 5A). These data confirm that the Ca^2+^ mobilization in response to BzATP in Figure 1 was not attributed to P2X_1_ receptor, a receptor potentially activated by BzATP. As we had evidence of non-canonical activity we asked if the cells expressed a variant form of the P2X_7_ receptor instead of or in addition to the full-length form. PCR was performed to amplify the 13 exons of P2X_7_ from genomic DNA extracted from HCLE cells. All exons were present (Figure 5B) and the specificity of the primers was confirmed by sequencing of the products. Although some single nucleotide polymorphisms were detected, none were reported to have effects on protein function (data not shown). Thus, the corneal epithelium has the ability to express a full-length transcript containing all exons. To further investigate, additional PCR was performed using primers that anneal to variants f and j [12]. As these variants lack exon 8, and the primers used span the deleted sequence, a product would not be detected if exon 8 was present (Figure 5C). The PCR product of the expected size was amplified and the product was sequenced and confirmed to be P2X_7_. The splicing of variant f does not lead to a C-terminal truncation but to a truncation of the N-terminus. Since BzATP causes enhanced pERK, a function of the N-terminus, it is unlikely that P2X_7f_ is the receptor being expressed in HCLE cells.

**Figure 6:**
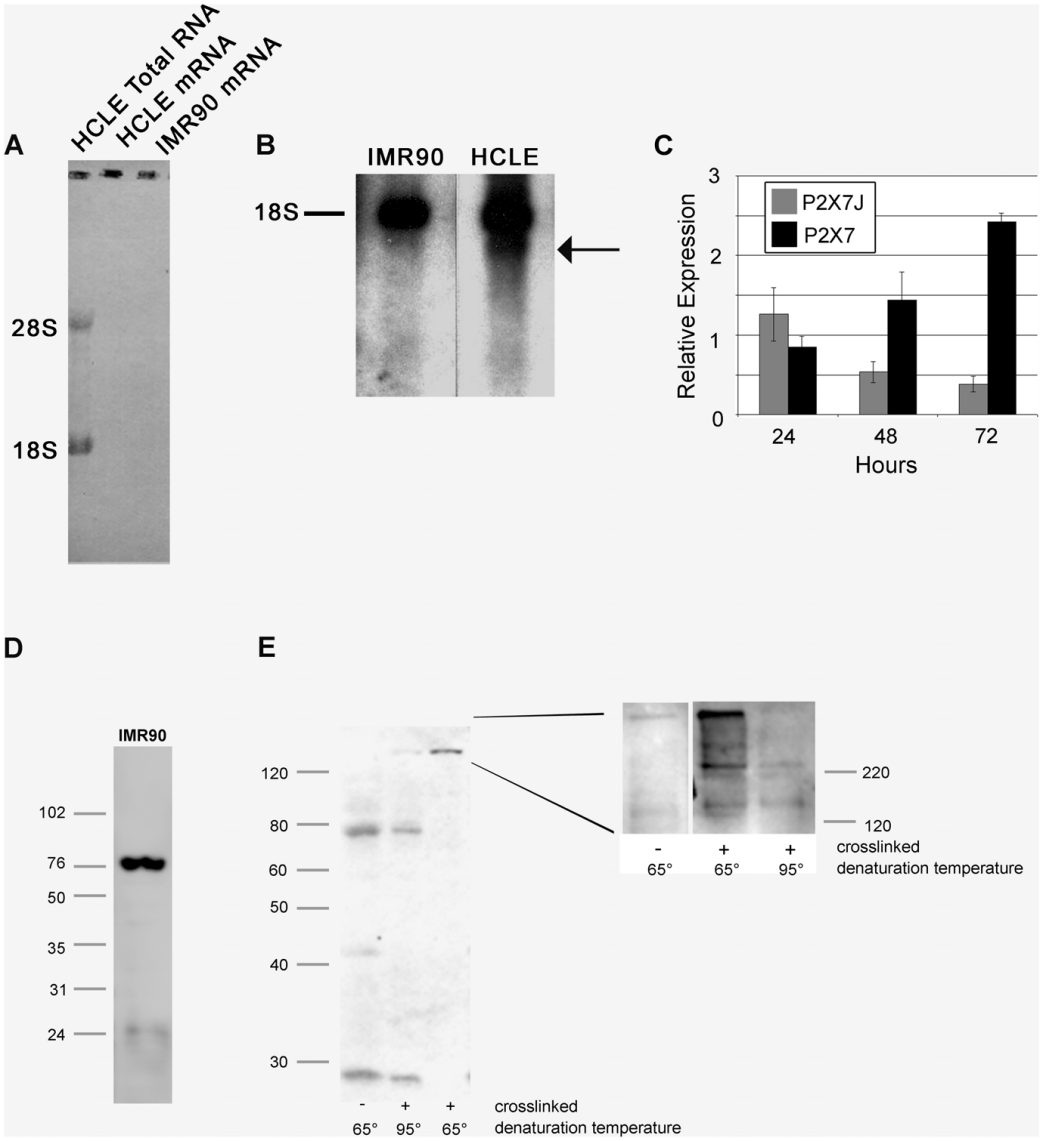
HCLEs express a full length and truncated P2X_7_ mRNA transcript and protein. **A.** mRNA was purified from total RNA of HCLE and IMR90 cells cultured for 72 hours by twice passing over an oligo-dT column. Purity was confirmed by methylene blue staining of nytran filter following transfer (note lack of rRNA in mRNA lanes). **B**. Northern blot analysis of HCLE and IMR90 mRNA from 72 hour cultures. The probe used was generated by PCR amplification of a consensus region in all P2X_7_ variants. The relative position of 18s rRNA is indicated and the smaller transcript expressed by epithelial cells is indicated (arrow). **C.** Expression of P2X_7_ and P2X_7j_ mRNA transcript over a 72 hour incubation. Relative expression was determined using the ΔΔCT method. **D.** Expression of P2X_7_ receptor by IMR90 cells. Cells were cultured for 72 hours, lysed and protein was resolved by SDS-PAGE (12%) and immunoblotted with anti-P2X_7_. **E.** Expression of P2X_7_ receptor by HCLE cells. Cells were cultured for 72 hours, lysed and protein was resolved by SDS-PAGE (12%) and immunoblotted with anti-P2X_7_. Hetero- and homotrimers are identified on crosslinking gels. Epithelial cells were cultured for 72 hours, incubated in situ in the presence of formaldehyde, lysed and incubated at 65°C or 95°C to maintain or break crosslinks. Inset – equivalent experiment resolved by SDS-PAGE (8%) and immunoblotted with anti-P2X_7_. Data is representative of a minimum of 3 experiments.

In additional experiments northern and western blot analysis revealed the presence of both full-length and variant forms of the P2X_7_ receptor. Northern blot analysis was performed on IMR90 cells (cells that exhibit large pore formation) and epithelial cells (Figure 6). Since the reported transcript sizes of several of the P2X_7_ receptor variants do not differ greatly from 18s rRNA, twice purified mRNA was used for the northern blot (Figure 6A). Ribosomal RNA was detected only in the total RNA fraction and was completely removed from the mRNA samples (Figure 6A). In the northern blot, both epithelial and IMR90 cells expressed a transcript of the same length; however, the epithelial cells expressed an additional transcript of a smaller size (Figure 6B). Previously established primer sequences were used to determine the expression of the j variant mRNA [12]. There were detectable changes in expression of the full-length and variant transcripts over time in HCLE cells (Figure 6C). In addition protein lysates from IMR90 cells and epithelial cells were examined for expression of P2X_7_ using western blot analysis and only the full-length 75 kDa form was detected in IMR90 cells (Figure 6D). In contrast, in HCLE cells the 75 kDa form was present in addition to a 42 kDa form, that we hypothesize corresponds to the j variant (Figure 6E). Specificity of the antibody to the P2X_7_ receptor was confirmed with non-immune rabbit IgG and peptide competition (data not shown).

The expression of the P2X_7_ multimers was examined using western blot analysis on cross-linked protein lysates. After treatment with formaldehyde the presence of a homotrimer of full length receptor (220 kDa) and heterotrimers along with homo- and heterodimers were detected at 65^o^C, and the 75 kDa form was minimal (Figure 6E). When the cross-linked lysates were brought to 95°C to break the crosslinks, the 75 kDa form is increased and there is a decrease in the higher molecular weight complexes (Figure 6E inset). Together these indicate that corneal epithelial cells have the ability to express both homo- and heterotrimers.

### Expression of P2X_7_ receptor is altered with stratification and disease

**Figure 7:**
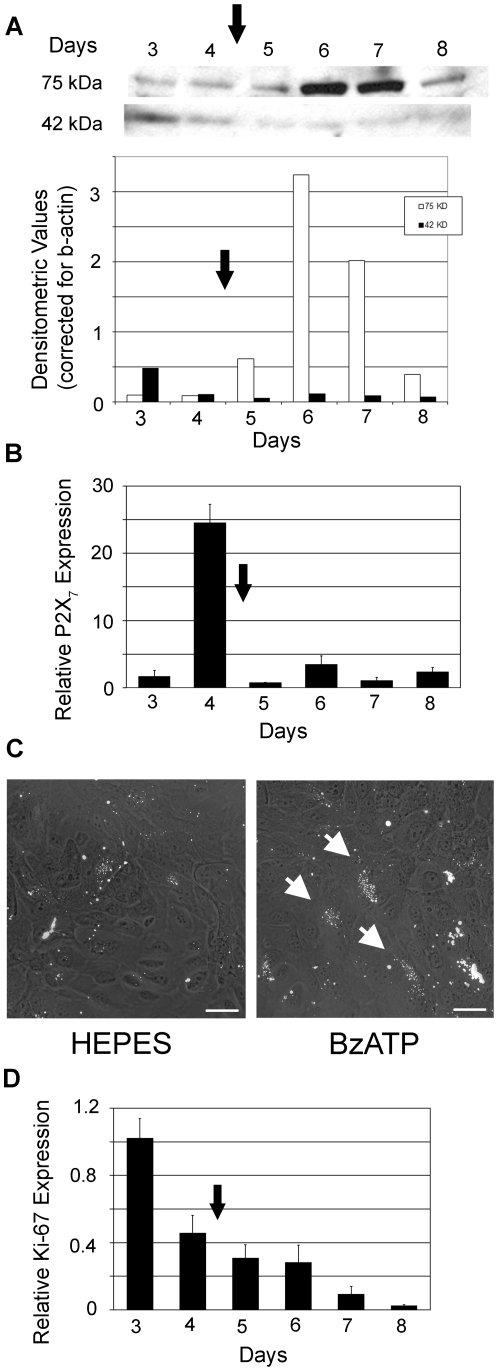
Expression of P2X_7_ receptor changes with stratification. **A.** Stratification was induced after day 4 (arrow) and cells were cultured for 4 additional days. Cells were lysed and equivalent amounts of protein were resolved by SDS-PAGE and immunoblotted with anti-P2X_7_. Representative blot is shown. Quantification of 75 kDa and 42 kDa protein are graphed as densitometric values relative to β-actin. Data is representative of a minimum of 4 independent experiments. **B.** Relative expression of P2X_7_ mRNA was determined using the ΔΔCT method and the average of 3 independent cultures +/− SEM is presented. **C.** Apical cells in stratified culture of HCLE cells at day 7 prestimulated with BzATP show minimal uptake of ToPro-3 using the flow through aparatus. Cultures were prestimulated with BzATP for 5 minutes at which time ToPro-3 in the presence of BzATP was added. Cells were imaged for an additional 20 minutes in the presence of BzATP as described in Figure 4 (scale bar  = 50 µm). Arrow indicates representative areas of uptake. Control experiments were performed in the presence of HEPES buffer. **D.** Relative expression of Ki67 mRNA was determined using the ΔΔCT method and the average of 3 independent cultures +/− SEM is presented.

To determine if there is a change in expression when cells were transitioned from a monolayer to stratified state, the expression of the P2X_7_ receptor was determined over 8 days. While we showed the presence of the full length and truncated forms in Figure 6E, we asked if the transition to stratification resulted in a change in the ratio of the forms. The 75 kDa P2X_7_ form increased after the stratification regime was initiated and remained elevated through day 7, compared to the 42 kDa form that decreased at 5 days and remained low (Figure 7A). A similar switch in the ratio of 75 and 42 kDa was confirmed in primary epithelial cells (data not shown). Expression was correlated to total P2X_7_ receptor mRNA over the same time period (Figure 7B). The increase in P2X_7_ mRNA was detected prior to the enhanced expression of the 75 kDa protein and decreased after stratification. The uptake of ToPro-3 was observed in some apical cells of the stratified cultures stimulated with BzATP (see arrows) compared to unstimulated and confluent cultures (Figure 7C). The uptake is correlated with the expression of the full length form and the decrease in the variant. In addition Ki67 mRNA, a marker of proliferation, decreased to negligible levels over the same time course (Figure 7D). Together, these indicate that as the cells stratify there is less proliferation and that the apical cells display uptake of the ToPro-3, consistent with an increase in expression of full length receptor.

**Figure 8:**
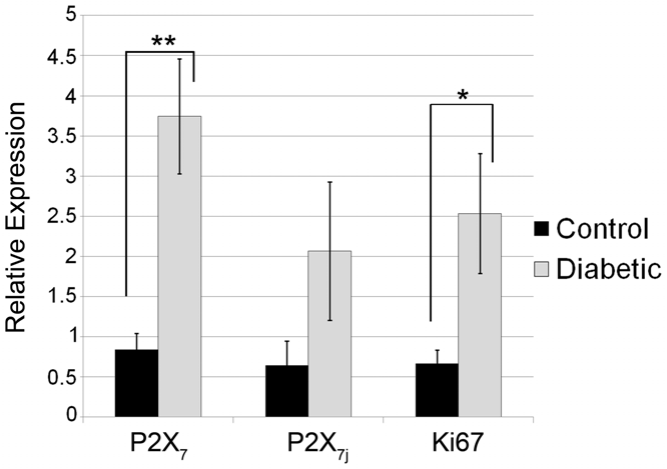
Diabetic corneal epithelium displays enhanced P2X_7_ receptor expression compared to control non-diabetic corneal epithelium. Central corneal epithelium from 6 samples of control and diabetic were analyzed using real time PCR. Relative expression of the P2X_7_ receptor (all variants), P2X_7j_ variant receptor, and Ki67 were determined using the ΔΔCT method. The average relative expression of 6 independent samples +/− SEM is presented. Significance was determined by Student's t-test: **p<0.002, *p<0.05 respectively.

To determine if expression of the P2X_7_ receptor was altered in a pathology in which wound repair and matrix organization is known to be altered, we examined diabetic corneal epithelium. The P2X_7_ receptor mRNA level was compared in age-matched diabetic and control non-diabetic corneas. The diabetic tissue showed a 4.4 fold increase in P2X_7_ mRNA over non-diabetic control that was significant (Figure 8). Furthermore there was an increased trend in expression of P2X_7j_ receptor mRNA. Unlike the control corneal epithelium the expression of pathologic tissue had a 3.8 fold increase in Ki67 mRNA.

## Discussion

Our results demonstrate that a normal healthy tissue such as corneal epithelium expresses one or more P2X_7_ splice variants. We demonstrate that the diabetic cornea displays significantly enhanced expression of P2X_7_ receptor and variant transcript compared to control. Our data indicate that the P2X_7_ receptor in the corneal epithelium is expressed as both full-length and variant forms and displays canonical and non-canonical activity. In Ca^2+^ mobilization experiments, BzATP induces a canonical response in control cultures in the presence of Mg^2+^ or Zn^2+^, or in the absence of extracelluar Ca^2+^. We demonstrated that P2X 4, 5, 6, and 7 receptor transcripts are expressed indicating that desensitization of the BzATP response following stimulation with ATP is not a P2X_1_ receptor mediated response since it is not present [4]. In addition, we have shown that as cells transition from monolayer to stratified the relative expression of the forms shift and there is detectable uptake of ToPro-3 only in the most apical cells. These indicate a functional change in phenotype as the confluent monolayer cultures do not take up the ToPro-3 in the presence or absence of BzATP. While previous studies have found variants in inflammatory or cancerous cells, it has not been shown in healthy tissue as a potential means for a single protein to modulate its cellular phenotype.

Previous studies have described polymorphisms of P2X_7_ and a number of mutations have been found in inflammatory cells [46], [47]. To date, eight variants resulting from alternative splicing have been identified [10], [48]. In the corneal epithelium all 13 exons of P2X_7_ from genomic DNA were found to be present along with some single nucleotide polymorphisms. None were synonymous with the known loss of function or gain of function mutations previously observed. As the corneal epithelium has the ability to express a full-length transcript containing all exons, additional PCR was performed using primers that anneal to variants f and j. Since these variants lack exon 8, and the primers that were used span the deleted sequence, a product would not be detected if exon 8 were maintained. Therefore the PCR product of the expected size was amplified and the resulting product was sequenced and confirmed to be P2X_7_. The region that was amplified contained a frameshift that is common to both f and j. The splicing of variant f does not lead to a C-terminal truncation but to a truncation of the N-terminus. Since BzATP causes enhanced pERK, a function of the N-terminus, it is unlikely that P2X_7f_ is the receptor being expressed in HCLE cells [10]. This is supported by data showing that the cells do not display cytotoxicity and confluent cells do not display dye uptake, which are all characteristics attributed to the carboxyl terminus. Together these data indicate that epithelial cells express a variant form of P2X_7_ receptor that we hypothesize is j.

The corneal epithelium is an excellent model tissue for studying the role of the P2X_7_ receptor and its variants. It is avascular, and renewal of the corneal epithelium occurs via movement of basal cells upward with shedding of the outermost epithelial layer into the tear fluid or in migration after injury. Since there were changes in the expression of truncated and full-length P2X_7_ receptor in epithelial cells during the transition from confluent monolayers to stratified cultures, we have begun to examine a number of pathologies, specifically epithelium from age matched diabetic and non-diabetic corneas. In diabetic tissue there are often changes in the adhesion of epithelium to the basal lamina and impaired wound repair [49]. We have shown altered wound repair and faulty epithelial adhesion in the P2X7^−/−^ mice [28]. While some studies indicate that the P2X_7_ receptor is associated with acceleration of Type I diabetes [50], other laboratories have hypothesized that apoptosis is defective in Type I diabetes [51]. Our results demonstrate that the diabetic cornea displays significantly enhanced expression of canonical P2X_7_ receptor compared to control along with an increase in Ki67 mRNA. These indicate that the variant mediates expression by acting as a dominant negative and may explain why others have found apoptosis to be defective in diabetes. Future studies will evaluate the presence of full-length and truncated forms along with the ability to form hetero- or homotrimers in normal and diseased tissue.

While the P2X_7j_ was detected in human cancerous cervical epithelial cells [11], [12], it is not unreasonable that it would be expressed in corneal epithelium. Investigators have shown that proteins change their regulation as the epithelium migrates to heal a wound and then again when it stratifies [52]. As we detect more of the 42 kDa form compared to the 75 kDa form in sparse cells and the inverse in stratified cells, the expression may depend on the state of epithelial integrity. In other words, the variant may provide the tissue flexibility as it migrates and becomes stratified during the process of wound closure and again as the cells move apically and shed. In addition the diabetic tissue displays a trend toward enhanced expression of the variant j compared to control. It is possible that the corneal epithelium expresses other variants and this will be a subject of future studies.

In other systems such as *Xenopus laevis*, the P2X_7_ receptor lacks the C-terminus and experiments have demonstrated that it can associate in varying ratios with the full-length receptor to form a heterotrimeric receptor with altered activity [53]. Still others have shown that P2X_7j_ hetero-oligomerizes with the full-length receptor resulting in antagonism of canonical activity [12]. Our physiological and biochemical data support this premise as our crosslinking studies indicate the presence of 220 kDa homotrimers along with heterotrimers containing truncated forms. Therefore, although the full-length receptor is expressed in the corneal epithelium, the presence of truncated subunits in the trimeric assembly of the receptor could inhibit the receptor's ability to form a large pore and induce a cytotoxic response. This is further supported by the work of Becker et al. who showed that lack of a single carboxyl tail in the trimeric P2X_7_ assembly is dominant-negative for receptor activity [53]. Together these data suggest a heterogeneous population of homo- and heterotrimeric receptors displaying a phenotype that resembles that of the heterotrimer.

The observation that corneal epithelial cells express a P2X_7_ variant may ensure that cell death does not regularly occur and disrupt corneal transparency and vision. While the epithelium maintains the ability to express the full-length receptor we hypothesize that in cell remodeling and/or pathology such as diabetes, that the balance is altered and the variant becomes upregulated. Further investigation into the expression and regulation of the P2X_7_ receptor should reveal an elegant mechanism for P2X_7_ to function as a multifaceted receptor that can mediate cell proliferation and migration or cell death.
